# Longitudinal default mode sub-networks in the language and visual variants of Alzheimer’s disease

**DOI:** 10.1093/braincomms/fcae005

**Published:** 2024-01-08

**Authors:** Irene Sintini, Nick Corriveau-Lecavalier, David T Jones, Mary M Machulda, Jeffrey L Gunter, Christopher G Schwarz, Hugo Botha, Arenn F Carlos, Michael G Kamykowski, Neha Atulkumar Singh, Ronald C Petersen, Clifford R Jack, Val J Lowe, Jonathan Graff-Radford, Keith A Josephs, Jennifer L Whitwell

**Affiliations:** Department of Radiology, Mayo Clinic, Rochester, MN 55905, USA; Department of Neurology, Mayo Clinic, Rochester, MN 55905, USA; Department of Radiology, Mayo Clinic, Rochester, MN 55905, USA; Department of Neurology, Mayo Clinic, Rochester, MN 55905, USA; Department of Psychiatry and Psychology, Mayo Clinic, Rochester, MN 55905, USA; Department of Radiology, Mayo Clinic, Rochester, MN 55905, USA; Department of Radiology, Mayo Clinic, Rochester, MN 55905, USA; Department of Neurology, Mayo Clinic, Rochester, MN 55905, USA; Department of Neurology, Mayo Clinic, Rochester, MN 55905, USA; Department of Information Technology, Mayo Clinic, Rochester, MN 55905, USA; Department of Neurology, Mayo Clinic, Rochester, MN 55905, USA; Department of Neurology, Mayo Clinic, Rochester, MN 55905, USA; Department of Radiology, Mayo Clinic, Rochester, MN 55905, USA; Department of Radiology, Mayo Clinic, Rochester, MN 55905, USA; Department of Neurology, Mayo Clinic, Rochester, MN 55905, USA; Department of Neurology, Mayo Clinic, Rochester, MN 55905, USA; Department of Radiology, Mayo Clinic, Rochester, MN 55905, USA

**Keywords:** atypical Alzheimer’s disease, default mode network, functional MRI, network failure quotient, tau

## Abstract

Disruption of the default mode network is a hallmark of Alzheimer’s disease, which has not been extensively examined in atypical phenotypes. We investigated cross-sectional and 1-year longitudinal changes in default mode network sub-systems in the visual and language variants of Alzheimer’s disease, in relation to age and tau. Sixty-one amyloid-positive Alzheimer’s disease participants diagnosed with posterior cortical atrophy (*n* = 33) or logopenic progressive aphasia (*n* = 28) underwent structural MRI, resting-state functional MRI and [^18^F]flortaucipir PET. One-hundred and twenty-two amyloid-negative cognitively unimpaired individuals and 60 amyloid-positive individuals diagnosed with amnestic Alzheimer’s disease were included as controls and as a comparison group, respectively, and had structural and resting-state functional MRI. Forty-one atypical Alzheimer’s disease participants, 26 amnestic Alzheimer’s disease participants and 40 cognitively unimpaired individuals had one follow-up functional MRI ∼1–2 years after the baseline scan. Default mode network connectivity was calculated using the dual regression method for posterior, ventral, anterior ventral and anterior dorsal sub-systems derived from independent component analysis. A global measure of default mode network connectivity, the network failure quotient, was also calculated. Linear mixed-effects models and voxel-based analyses were computed for each connectivity measure. Both atypical and amnestic Alzheimer’s disease participants had lower cross-sectional posterior and ventral and higher anterior dorsal connectivity and network failure quotient relative to cognitively unimpaired individuals. Age had opposite effects on connectivity in Alzheimer’s disease participants and cognitively unimpaired individuals. While connectivity declined with age in cognitively unimpaired individuals, younger Alzheimer’s disease participants had lower connectivity than the older ones, particularly in the ventral default mode network. Greater baseline tau-PET uptake was associated with lower ventral and anterior ventral default mode network connectivity in atypical Alzheimer’s disease. Connectivity in the ventral default mode network declined over time in atypical Alzheimer’s disease, particularly in older participants, with lower tau burden. Voxel-based analyses validated the findings of higher anterior dorsal default mode network connectivity, lower posterior and ventral default mode network connectivity and decline in ventral default mode network connectivity over time in atypical Alzheimer’s disease. Visuospatial symptoms were associated with default mode network connectivity disruption. In summary, default mode connectivity disruption was similar between atypical and amnestic Alzheimer’s disease variants, and discriminated Alzheimer’s disease from cognitively unimpaired individuals, with decreased posterior and increased anterior connectivity and with disruption more pronounced in younger participants. The ventral default mode network declined over time in atypical Alzheimer’s disease, suggesting a shift in default mode network connectivity likely related to tau pathology.

## Teaser

Sintini *et al*. report that atypical Alzheimer’s disease patients have lower posterior and ventral and higher anterior dorsal default mode network connectivity and network failure quotient relative to cognitively unimpaired individuals, similarly to the amnestic variant. Tau pathology correlated with lower ventral default mode network connectivity, which declined over time.

## Graphical Abstract

**Graphical Abstract:**
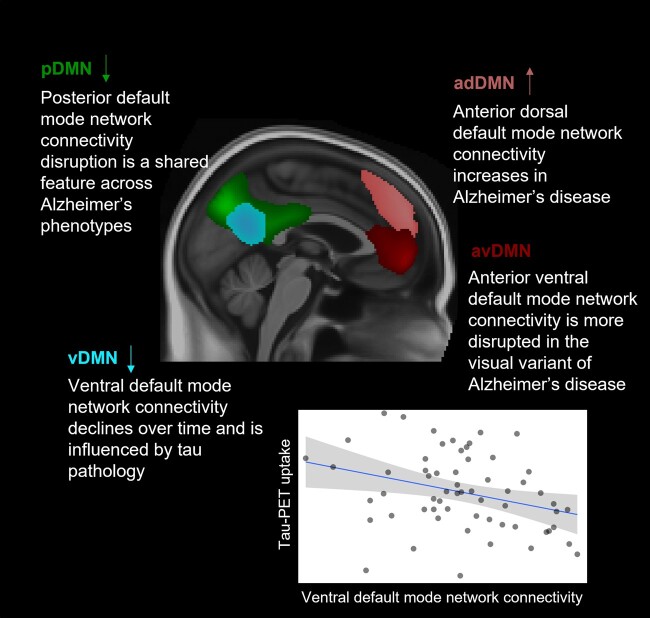

## Introduction

Disruption of large-scale brain networks accompanies normal aging and is implicated in cognitive decline even in absence of neurodegenerative disease.^1^ Changes in episodic memory have been associated with changes in the functional connectivity of the default mode network (DMN), even after adjusting for atrophy, in cognitively normal older adults.^2^ Functional alteration of the DMN has been used to predict cortical thinning independently of temporal tau burden in preclinical Alzheimer’s disease.^3^ Among large-scale brain networks, the DMN is particularly relevant in Alzheimer’s disease. The DMN overlaps with the spatial patterns of amyloid-β deposition,^4^ and the alterations in its functional connectivity that characterize healthy aging are accelerated in the presence of Alzheimer’s disease.^5,6^ Therefore, in dementia caused by Alzheimer’s disease, cognitive decline likely derives not only from loss of neurons but also from the abnormal use of the remaining neurons, which is evident on glucose metabolism PET and functional MRI (fMRI). Transcranial magnetic stimulation of the precuneus, which is a key node of the DMN, decelerated cognitive decline in Alzheimer’s disease in a randomized trial.^7^

Besides the precuneus, the DMN encompasses regions in the medial temporal lobe, posterior cingulate cortex, anterior and dorsal medial prefrontal cortex, and it is fractioned into sub-systems active during different mental states.^8^ Independent component analyses of blood oxygenation level–dependent signal from resting-state fMRI of cognitively normal individuals demonstrated that the DMN is made up of an anterior dorsal (adDMN) and anterior ventral (avDMN) component, a posterior component (pDMN) and a ventral component (vDMN).^9,10^ A breakdown in connectivity between the anterior and posterior components and reduced posterior connectivity characterize aging and Alzheimer’s disease.^5^ These measures have been summarized in the network failure quotient (NFQ),^11,12^ which increases with age and disease severity. This biomarker of DMN changes is robust across normal aging and Alzheimer’s disease amnestic and dysexecutive variants.^6^

Across Alzheimer’s disease phenotypes, tau-associated disruption of phenotype-specific networks can cause amyloid-associated phenotype-independent compensatory changes in the DMN.^10^ Although the spatial pattern of the DMN does not include phenotype-specific regions of Alzheimer’s atypical variants, analyses of DMN connectivity in atypical non-amnestic variants of Alzheimer’s disease showed mixed results, likely due to heterogeneous methodologies and patients’ characteristics. Increased functional connectivity in the anterior component of the DMN has been reported in the visual and language variants,^13^ similarly to amnestic Alzheimer’s disease,^5^ while the posterior component was not abnormal.^13^ Connectivity in vDMN was not altered in the language variant,^14^ which also exhibited areas of enhanced connectivity within the occipital regions of the DMN.^15^ In the visual variant, DMN connectivity ranged from indistinguishable from normal controls^16^ to weaker^17^ or increased.^18^ Tau pathology in the posterior cingulate cortex has been related to disruption of the DMN connectivity within its posterior parietal regions in the language, visual and executive variants of Alzheimer’s disease.^19^

Cross-sectional analyses of the age effect on DMN connectivity shed light on the changes that happen over the lifespan in healthy aging and Alzheimer’s disease. Longitudinal analyses can provide a more accurate picture of the pathophysiological mechanisms that drive disease progression, although longitudinal fMRI measures pose challenges for their low intra-individual repeatability and high inter-individual variability.^20^ In Alzheimer’s disease, longitudinal changes were detected within the anterior and posterior DMN connectivity, which was reduced over a 2–4-year interval relative to healthy aging, even though the anterior component of the network was more activated at baseline in the disease group.^21^ Amyloid-β burden has been associated with faster longitudinal disruption of the default mode and salience network axis in Alzheimer’s disease.^22^ In cognitively normal older adults, connectivity within the DMN decreased over time,^2,23^ and the decline had a non-linear trajectory, with individuals over 74 years old experiencing an acceleration in connectivity decline.^2^ In contrast, one study failed to identify longitudinal connectivity changes within the DMN in healthy older adults over 6 years.^24^ No studies have investigated longitudinal changes in the DMN in atypical Alzheimer’s disease variants.

The objective of this study was to investigate cross-sectional and longitudinal DMN connectivity within its adDMN, avDMN, pDMN and vDMN components, in participants diagnosed with the language or visual variants of Alzheimer’s disease relative to age-matched cognitively unimpaired (CU) individuals and to individuals diagnosed with the amnestic variant of Alzheimer’s disease. Secondary objectives of this study were to investigate the effect of age on DMN connectivity changes over time, as younger age is typically associated with a more aggressive disease course,^25-27^ as well as the effect of tau on such changes in atypical Alzheimer’s disease, as tau has been shown to correlate with disrupted functional connectivity.^19,28,29^ In addition to increasing our knowledge of the pathophysiology of the visual and language variants of Alzheimer’s disease, investigating DMN connectivity patterns in these two phenotypes will help further clarify whether DMN connectivity changes are a biomarker of Alzheimer’s disease shared across clinical phenotypes, as postulated by the cascading network failure model. Lastly, measuring DMN connectivity within the cascading network failure hypothesis may help bring together divergent findings from previous studies on atypical variants of Alzheimer’s disease conducted within heterogeneous frameworks.

## Materials and methods

### Participants

Sixty-one atypical Alzheimer’s disease participants were included in the study. Thirty-three participants met clinical criteria for posterior cortical atrophy (PCA),^30^ i.e. the visual variant of Alzheimer’s disease, and 28 met clinical criteria for logopenic progressive aphasia (LPA),^31^ i.e. the language variant of Alzheimer’s disease. Participants were recruited by the Neurodegenerative Research Group (NRG; PI’s Whitwell and Josephs) between 2016 and 2020 and underwent structural MRI and resting-state fMRI, [^11^C]Pittsburgh compound B (PiB) PET for amyloid-β, and [^18^F]flortaucipir PET for tau. Forty-one participants (20 PCA and 21 LPA) also underwent 1-year follow-up structural and fMRI. The Aβ-PET scans were analysed to determine Aβ positivity as previously described,^32^ and all participants were determined to be Aβ(+) at baseline except for one, who converted to positivity at follow-up. All participants exhibited flortaucipir uptake patterns consistent with their Alzheimer’s disease clinical phenotype. All participants underwent a clinical and neuropsychological evaluation including the Montreal Cognitive Assessment (MoCA)^33^ to assess general cognitive function, the Clinical Dementia Rating Sum of Boxes (CDR-SB)^34^ to assess functional ability, the 15-item Boston Naming Test (BNT)^35^ to assess confrontational naming, the Boston Diagnostic Aphasia Examination (BDAE)^36^ repetition sub-test for sentence repetition, the Visual Object and Space Perception (VOSP) incomplete letter and cube tests^37^ to assess visuoperceptual and visuospatial functioning, respectively, and the Rey–Osterrieth (Rey–O) Complex Figure Copy^38^ to assess visuoconstruction. The severity of simultanagnosia was determined on a 20-point scale with 20 being the best score. Apolipoprotein E (APOE) genotyping was performed on 59 participants. Atypical Alzheimer’s disease participants were age and sex matched 1:2 to 122 CU (CDR-SB = 0), Aβ(−) participants from the Mayo Clinic Study of Aging (MCSA),^39^ who completed the same imaging protocol. Forty out of 122 CU individuals underwent follow-up imaging, and all remained Aβ(−) at follow-up. Atypical Alzheimer’s disease participants were also age and sex matched 1:1 to 61 Aβ(+) participants diagnosed with amnestic Alzheimer’s disease from the MCSA or the Alzheimer’s Disease Research Center (ADRC), who completed the same imaging protocol. One patient was excluded because their fMRI was unusable. Twenty-six out of the final 60 amnestic Alzheimer’s disease participants underwent follow-up imaging. CU individuals and amnestic Alzheimer’s disease participants were evaluated clinically with the CDR-SB and the Mini-Mental State Examination (MMSE),^40^ as per the clinical protocol of MCSA and ADRC. MMSE scores for atypical Alzheimer’s disease participants were obtained converting their MoCA scores.^41^ The study was approved by the Mayo Clinic IRB, and all participants provided written informed consent to participate in this study.

### Image acquisition

PET scans were acquired using PET/CT scanners (GE Healthcare, Milwaukee, WI, or Siemens Healthcare, Erlangen, Germany) operating in 3D mode. For tau-PET, an intravenous bolus injection of ∼370 MBq (range 333–407 MBq) of [^18^F]flortaucipir was administered, followed by a 20-min PET acquisition performed 80 min after injection. For Aβ-PET, participants were injected with PiB of ∼628 MBq (range 385–723 MBq) and, after a 40–60-min uptake period, a 20-min PiB scan was obtained. All PET scans consisted of four 5-min dynamic frames following a low-dose CT transmission scan. Standard corrections were applied. Emission data were reconstructed into a 256 × 256 matrix with a 30 cm field of view (in-plane pixel size = 1.0 mm). All participants also underwent a 3 T head MRI performed on GE scanners (GE Healthcare, Milwaukee, WI). The MRI protocol included a magnetization prepared rapid gradient echo (MPRAGE) sequence (TR/TE/TI, 2300/3/900 ms; flip angle 8°, 26 cm field of view, 256 × 256 in-plane matrix with a phase field of view of 0.94 and slice thickness of 1.2 mm)^42^ and resting-state gradient echo-planar imaging (TR/TE = 3000/30 ms, 90° flip angle, slice thickness 3.3 mm, in-plane resolution 3.3 mm and 160 volumes). Participants were instructed to keep their eyes open during the resting-state fMRI scanning.

#### Structural MRI and PET preprocessing

Each MPRAGE was segmented and bias field corrected using Unified Segmentation^43^ in SPM12 (Wellcome Trust Centre for Neuroimaging, London, UK), with Mayo Clinic Adult Lifespan Template (MCALT; https://www.nitrc.org/projects/mcalt/) tissue priors and settings.^44^ Using ANTs,^45^ the MCALT atlas was propagated to the native MPRAGE space for region-level measurements. PET images were rigidly registered to the corresponding MPRAGE using SPM12. PET median regional standard uptake value ratios (SUVR) were then calculated in voxels segmented as grey or white matter with the cerebellar crus grey matter as the reference region. Tau-PET SUVR was calculated in a meta-region of interest (ROI) specific for atypical Alzheimer’s disease, including parietal (inferior and superior parietal, supramarginal, angular and precuneus), temporal (inferior, middle and superior temporal) and occipital (inferior, middle and superior occipital) regions of the MCALT atlas.

#### fMRI preprocessing

fMRI were processed as previously described.^10,46,47^ The fMRI sequences of the participants included in the study had fewer than 3 mm of translational movement and fewer than 3° of rotational movement, without evidence of obvious artefacts on visual inspection. At baseline imaging, maximum head movement differed between atypical Alzheimer’s disease participants and amnestic Alzheimer’s disease participants and CU individuals on two-sample *t*-tests [median values for atypical Alzheimer’s disease participants, amnestic Alzheimer’s disease and CU individuals] (*x* mm: 0.2, 0.2, 0.2, *P*_atypical Alzheimer’s disease CU_ = 0.01, *P*_atypical Alzheimer’s disease amnestic Alzheimer’s disease_ = 0.4; *y* mm: 0.3, 0.3, 0.3, *P*_atypical Alzheimer’s disease CU_ = 0.04, *P*_atypical Alzheimer’s disease amnestic Alzheimer’s disease_ = 0.4; *z* mm: 0.6, 0.6, 0.7, *P*_atypical Alzheimer’s disease CU_ = 0.7, *P*_atypical Alzheimer’s disease amnestic Alzheimer’s disease_ = 0.3; roll degrees: 0.4, 0.3, 0.3, *P*_atypical Alzheimer’s disease CU_ = 0.2, *P*_atypical Alzheimer’s disease amnestic Alzheimer’s disease_ = 0.5; pitch degrees: 0.6, 0.6, 0.7, *P*_atypical Alzheimer’s disease CU_ = 0.7, *P*_atypical Alzheimer’s disease amnestic Alzheimer’s disease_ = 0.1; yaw degrees: 0.4, 0.3, 0.4, *P*_atypical Alzheimer’s disease CU_ = 0.008, *P*_atypical Alzheimer’s disease amnestic Alzheimer’s disease_ = 0.3; frame-wise displacement: 0.2, 0.2, 0.2, *P*_atypical Alzheimer’s disease CU_ = 0.6, *P*_atypical Alzheimer’s disease amnestic Alzheimer’s disease_ = 0.2). Atypical Alzheimer’s disease participants had higher maximum motion in the *x*- and *y*-directions and higher maximum yaw rotation than CU individuals. The spatial temporal regression process resulted in participant-specific spatial maps of each DMN sub-system, i.e. adDMN, avDMN, pDMN and vDMN high-dimensional independent components of the MCSA Functional Connectivity Atlas. Only voxels with probability of being grey matter >0.5 were included to avoid potential bias related to differences in grey matter volume between Alzheimer’s disease and CU participants. In the maps, each voxel value is the correlation between that voxel time course and the independent component time course (i.e. the network defined with independent component analysis). These maps were then transformed into *z*-score maps. Within-network connectivity was calculated as the median *z*-score of the voxels within each sub-system, in the participant-specific space. Between-network connectivity was extracted as the correlation between the time courses in each sub-systems pair to calculate the NFQ as in (1).


(1) $$\mathrm{NFQ}=\;\frac{\mathrm{vDMN}\_\mathrm{to}\_\mathrm{pDMN}\;+\;\mathrm{adDMN}\_\mathrm{to}\_\mathrm{pDMN}}{\mathrm{pDMN}\;+\;\mathrm{vDMN}}.$$

Participant-specific spatial maps of the four sub-networks were then transformed into the MCALT template space and smoothed with 4 mm full-width half maximum Gaussian kernel for voxel-level analyses.

### Statistical analysis

Baseline characteristics were compared between the participants’ groups with Fisher’s exact test for categorical variables and Wilcoxon rank-sum test for continuous variables.

#### Mixed-effects models

Longitudinal linear mixed-effects models were fit separately for each functional connectivity quantity (2).


(2) connectivity∼sex+APOEε4+dx+dx:age+dx:time+dx:time:age+(1|participant).

The response variables were each sub-network’s connectivity *z*-score and the NFQ. The regression parameters were age at the time of the first scan, diagnosis (dx) of either atypical Alzheimer’s disease, amnestic Alzheimer’s disease or CU status, time and the interactions between time and diagnosis; age and diagnosis; and age and time and diagnosis. Time was expressed as years from first scan and had an origin of zero. APOE ɛ;4 status and sex were also included as fixed effects. Models were also re-ran including the fMRI maximum head motions as fixed effects to ensure that connectivity differences between Alzheimer’s disease participants and CU individuals were not due to motion differences. The regression parameter of primary interest was the interaction between time and diagnosis, which corresponds to the mean difference in annual rates of change in DMN sub-system connectivity between CU and Alzheimer’s disease participants. The models were run with age centred at 55, 65 and 75 years. The models were generated using all cross-sectional and longitudinal data points for each participant. The models included subject-specific random intercepts. The same models were run including only atypical Alzheimer’s disease participants and defining the diagnosis as PCA or LPA, to investigate differences between the two atypical phenotypes.

To investigate the effect of tau-PET SUVR on DMN connectivity, baseline connectivity *z*-scores of all four sub-networks and the NFQ were assessed against baseline tau-PET SUVR in the atypical Alzheimer’s disease meta-ROI with separate linear regression models. Mixed-effects models were also fit on each sub-network’s connectivity *z*-score and the NFQ to investigate the effect of baseline tau on DMN connectivity cross-sectionally and longitudinally in atypical Alzheimer’s disease (3).


(3) connectivity∼sex+age+time+Aß+tau+tau:time+(1|participant).

These models included only atypical Alzheimer’s disease participants (*n* = 61). The regression parameters of the mixed-effects models were age at the time of the first scan, sex, log of baseline global Aβ SUVR, time, log of baseline tau-PET SUVR in the atypical Alzheimer’s disease meta-ROI and the interaction between time and tau. The regression parameters were summarized from each model in terms of point estimates and 95% confidence intervals (CIs) displayed in figures. Ninety-five percent confidence intervals that do not include zero correspond to a significant effect at *P* < 0.05. All mixed-effects models were fit with the lmer function in the lme4 package in R version 4.2.2.

#### Voxel-based analyses

The voxel-wise connectivity map of each default mode sub-network was obtained for atypical Alzheimer’s disease participants and CU individuals with a one-sample *t*-test for visualization purposes. Multiple regression analyses were performed on the images representing each sub-network’s connectivity to compare atypical (*n* = 59) Alzheimer’s disease participants to CU individuals (*n* = 122), covarying for age, sex and APOE ɛ;4 status. Analyses were also repeated comparing CU individuals to PCA and LPA separately, including two age- and sex-matched CU individuals among the ones previously selected for each PCA (*n* = 32) or LPA (*n* = 27) participant. Paired *t*-tests between baseline and follow-up images were performed separately on atypical Alzheimer’s disease participants (*n* = 41) and CU individuals (*n* = 40) to investigate connectivity changes over time. Results were masked with each sub-network ROI from the MCSA Functional Connectivity Atlas and visualized with BrainNet Viewer.^48^ Analyses were performed in SPM12.

#### Clinical connectivity correlations

Baseline connectivity *z*-scores of all four sub-networks and the NFQ were assessed against clinical scores in atypical and amnestic Alzheimer’s disease participants with separate linear regression models. In each model, the connectivity was used as the predictor and the clinical score as the outcome. The included clinical scores were MMSE, CDR-SB, MoCA, BNT, BDAE repetition, VOSP letters, VOSP cubes, Rey–O Complex Figure Copy and simultanagnosia.

## Results

### Participants

Demographics and clinical characteristics are reported in Table 1. Atypical Alzheimer’s disease participants had a higher prevalence of APOE ɛ;4 carriers than CU individuals (*P* < 0.001), but a lower prevalence than amnestic Alzheimer’s disease participants (*P* = 0.003). PCA participants were younger (*P* = 0.03) and had a longer disease duration than LPA (*P* = 0.01). Atypical Alzheimer’s participants had shorter disease duration than the amnestic participants (*P* < 0.001), although disease duration was only known for 16 out of 60 of the amnestic participants. By design, atypical Alzheimer’s disease participants had higher global Aβ SUVR than CU individuals (*P* < 0.001) and performed worse on CDR-SB (*P* < 0.001) and MMSE (*P* < 0.001). Amnestic Alzheimer’s disease participants performed worse than the atypical participants on CDR-SB (*P* = 0.002) and MMSE (*P* = 0.003). PCA performed worse on CDR-SB (*P* = 0.04), Rey–O (*P* < 0.001), test of simultanagnosia (*P* < 0.001), VOSP letters (*P* < 0.001) and VOSP cubes (*P* < 0.001) than LPA and LPA performed worse on BDAE repetition than PCA (*P* = 0.02). The time difference between baseline and follow-up MRI was shorter in atypical Alzheimer’s participants than CU individuals (*P* < 0.001) and amnestic participants (*P* = 0.04). This discrepancy was due to differences in recruitment strategies between NRG and MCSA, ADRC and to limited availability of individuals with longitudinal fMRI in MCSA and ADRC.

**Table 1:** Clinical and demographic characteristics

	CU	Atypical Alzheimer’s disease			Amnestic Alzheimer’s disease	*P*-value		
		PCA + LPA	PCA	LPA		Atypical Alzheimer’s disease versus CU	PCA versus LPA	Atypical Alzheimer’s disease versus amnestic Alzheimer’s disease
*N*	122	61	33	28	60			
Demographics								
Male (*n*)	44 (36%)	22 (36%)	12 (36%)	10 (36%)	22 (37%)	1	1	1
Education	15 (2.5)	16 (2.4)	16 (3)	16 (2)	16 (3)	0.2	0.5	0.7
Age	65 (7)	65 (7)	62 (7)	67 (7)	65 (7)	0.8	0.03^a^	0.8
Disease duration		3.3 (2.5)	4.2 (2.7)	2.4 (1.9)	6.2 (2.6)		0.01^a^	<0.001^a^
APOE ɛ;4 carriers (*n*)	21 (17%)	25 (42%)	16 (50%)	9 (33%)	42 (71%)	<0.001^a^	0.3	0.003^a^
Imaging quantities								
Global PiB SUVR at baseline	1.35 (0.06)	2.4 (0.4)	2.4 (0.3)	2.4 (0.5)	2.5 (0.4)	<0.001^a^	0.9	0.2
Atypical Alzheimer’s disease meta-ROI tau SUVR at baseline		2.2 (0.5)	2.2 (0.5)	2.1 (0.4)	2.1 (0.5)		0.1	0.8
Temporal meta-ROI tau SUVR at baseline		2.1 (0.4)	2.1 (0.5)	2.1 (0.3)	2.1 (0.4)		0.9	0.8
Neurological tests								
MoCA		18 (6.4)	17 (6)	18.5 (6.9)			0.8	
MMSE	29 (1)	24 (7)	22 (8)	25 (7)	20 (5)	<0.001^a^	0.5	0.003^a^
CDR-SB	0 (0)	3 (4)	3 (4)	2 (3)	5 (3)	<0.001^a^	0.04^a^	0.002^a^
Neuropsychological tests								
Simultanagnosia		13 (7)	7 (6)	19 (2)			<0.001^a^	
BNT		11.5 (3)	12 (3)	11 (4)			0.1	
BDAE repetition		8 (2)	8 (2)	7 (2)			0.02	
VOSP letters		17.5 (7)	13 (7)	20 (2)			<0.001^a^	
VOSP cubes		4 (4)	1 (3)	9 (3)			<0.001^a^	
Rey–O Complex Figure MOANS		2 (3)	2 (1)	6 (4)			<0.001^a^	
Participants with follow-up visit								
*N*	40	41	20	21	26			
Years difference between MRI	2.1 (0.5)	1.0 (0.1)	1 (0.1)	0.99 (0.5)	1.05 (0.5)	<0.001^a^	0.4	0.04^a^

Data are shown as *n* (%) or median (standard deviation). For continuous variables, *P*-values are from Wilcoxon rank-sum test. For categorical variables, *P*-values are from Fisher’s exact test. APOE status was not available for three participants (one PCA, one LPA and one amnestic Alzheimer’s disease). Disease duration was only known for 16 amnestic Alzheimer’s disease participants. Tau-PET was only available for 39 amnestic Alzheimer’s disease patients. MMSE scores for atypical Alzheimer’s disease participants were obtained converting their MoCA scores. CU, cognitively unimpaired; PCA, posterior cortical atrophy; LPA, logopenic progressive aphasia; APOE, apolipoprotein E; MoCA, Montreal Cognitive Assessment; MMSE, Mini-Mental State Examination; CDR-SB, Clinical Dementia Rating Sum of Boxes; BNT, 15-item Boston Naming Test; BDAE, Boston Diagnostic Aphasia Examination; VOSP, Visual Object and Space Perception; Rey–O Complex Figure MOANS, Rey–Osterrieth Complex Figure Copy Mayo’s Older Americans Normative Studies.

^a^Significantly different.

### Mixed-effects models

#### Atypical and amnestic Alzheimer’s disease participants versus CU individuals

**Figure 1:**
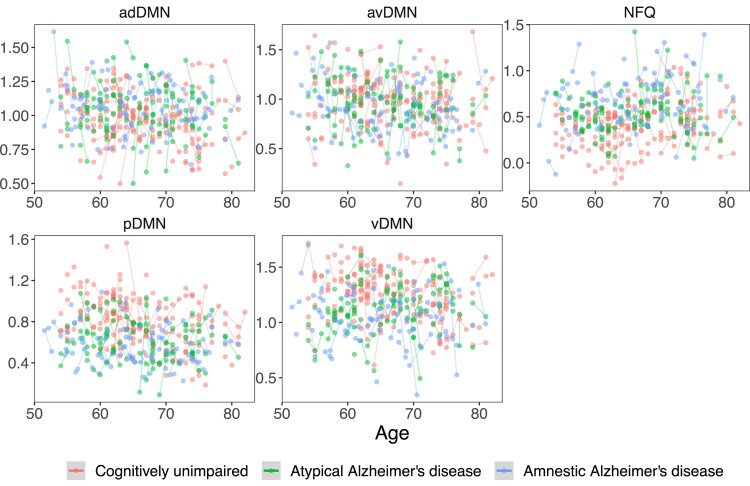
**Functional connectivity values.** Raw functional connectivity values for adDMN, avDMN, pDMN, vDMN and NFQ. adDMN, anterior dorsal default mode network; avDMN, anterior ventral default mode network; pDMN, posterior default mode network; vDMN, ventral default mode network; NFQ, network failure quotient.

**Figure 2:**
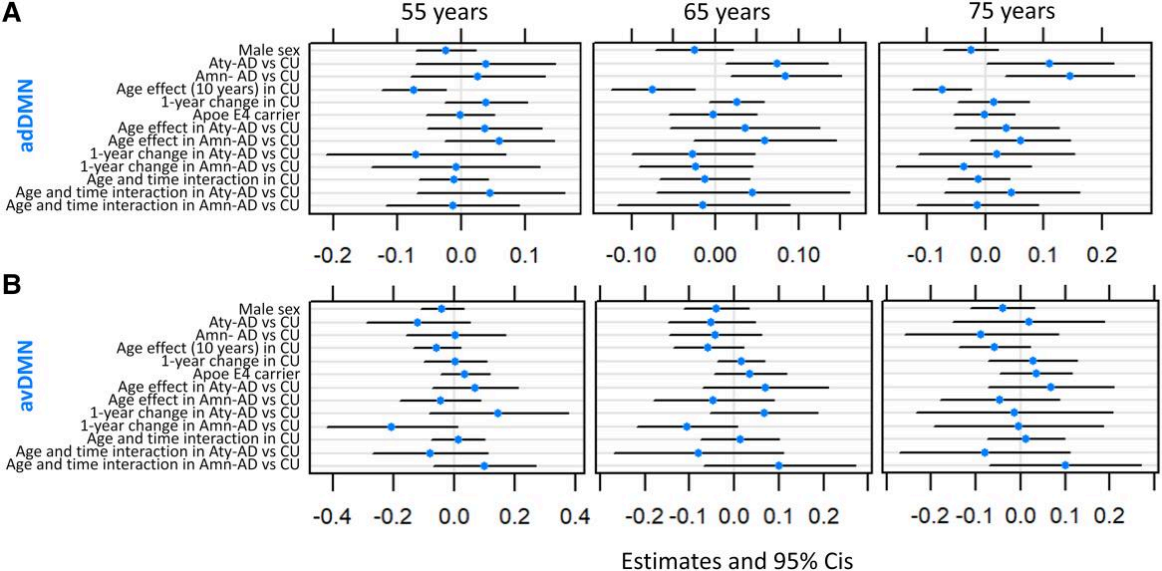
**Mixed-effects models of adDMN and avDMN in atypical and amnestic Alzheimer’s disease.** Mixed-effects models estimates for adDMN (**A**) and avDMN (**B**). Models are run including only participants with known APOE ɛ;4 status: 59 atypical Alzheimer’s disease (39 of which with follow-up data), 59 amnestic Alzheimer’s disease (26 of which with follow-up data) and 122 CU individuals (40 of which with follow-up data). Results are reported with age centred at 55, 65 and 75 years. The age and time interaction can be interpreted as the 1-year change effect modifier for 10 years of age. All fixed effects are reported. Ninety-five per cent confidence intervals that do not cross the 0 line represent significant effects at the *P* < 0.05 level. Aty-AD, atypical Alzheimer’s disease; Amn-AD, amnestic Alzheimer’s disease; CU, cognitively unimpaired; adDMN, anterior dorsal default mode network; avDMN, anterior ventral default mode network; pDMN, posterior default mode network; vDMN, ventral default mode network; NFQ, network failure quotient; APOE, apolipoprotein.

**Figure 3:**
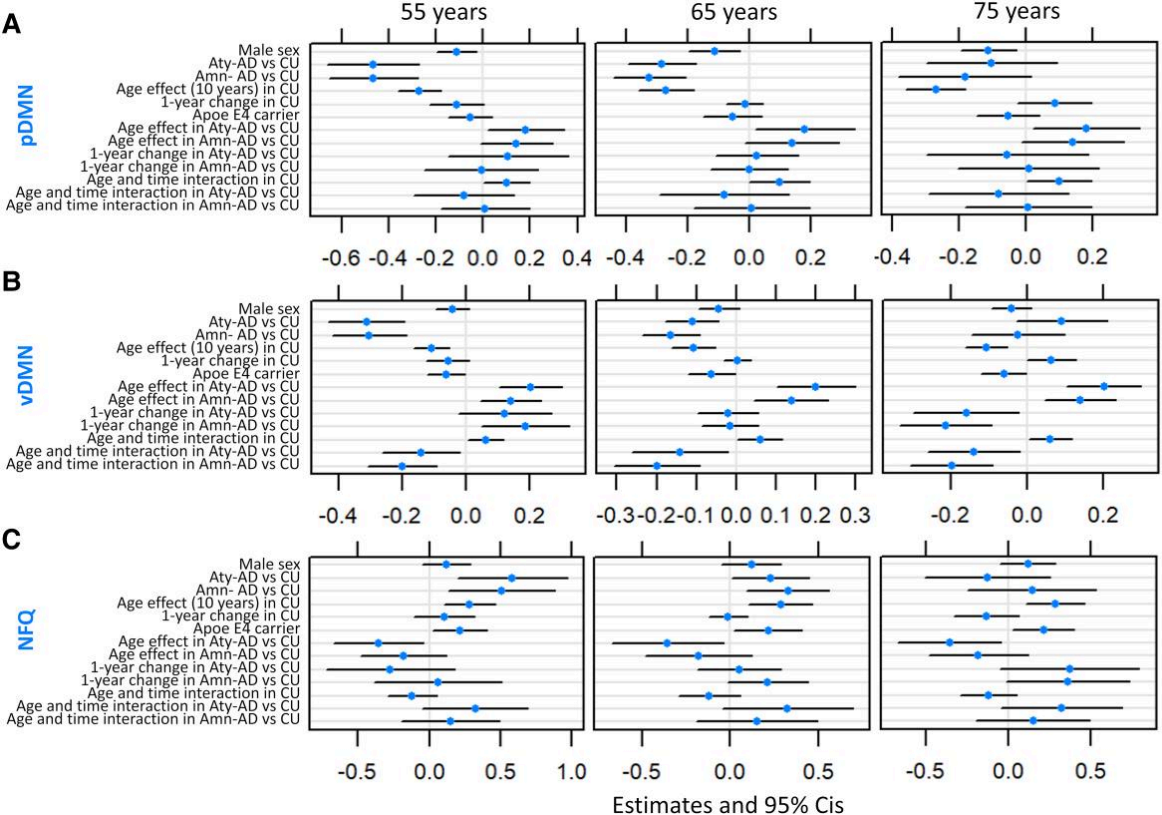
**Mixed-effects models of pDMN, vDMN and NFQ in atypical and amnestic Alzheimer’s disease.** Mixed-effects model estimates for pDMN (**A**), vDMN (**B**) and NFQ (**C**). Models are run including only participants with known APOE ɛ;4 status: 59 atypical Alzheimer’s disease (39 of which with follow-up data), 59 amnestic Alzheimer’s disease (26 of which with follow-up data) and 122 CU individuals (40 of which with follow-up data). Results are reported with age centred at 55, 65 and 75 years. The age and time interaction can be interpreted as the 1-year change effect modifier for 10 years of age. All fixed effects are reported. Ninety-five per cent confidence intervals that do not cross the 0 line represent significant effects at the *P* < 0.05 level. Aty-AD, atypical Alzheimer’s disease; Amn-AD, amnestic Alzheimer’s disease; CU, cognitively unimpaired; adDMN, anterior dorsal default mode network; avDMN, anterior ventral default mode network; pDMN, posterior default mode network; vDMN, ventral default mode network; NFQ, network failure quotient; APOE, apolipoprotein.

Raw functional connectivity values used in the mixed-effects models are shown in Fig. 1. At 65 years of age, adDMN connectivity was significantly higher in atypical (*β* = 0.07, *P* = 0.02) and amnestic (*β* = 0.08, *P* = 0.01) Alzheimer’s disease participants than in CU individuals (Fig. 2A). Conversely, at 65 years of age, pDMN and vDMN connectivity was significantly lower and the NFQ was significantly higher in atypical (pDMN: *β* = −0.28, *P* < 0.001, vDMN: *β* = −0.11, *P* = 0.004, NFQ: *β* = 0.23, *P* = 0.05) and amnestic (pDMN: *β* = −0.32, *P* < 0.001, vDMN: *β* = −0.16, *P* < 0.001, NFQ: *β* = 0.33, *P* = 0.006) Alzheimer’s disease participants than in CU individuals (Fig. 3). Among CU individuals, older age had a positive effect on NFQ (*β* = 0.28, *P =* 0.004) and a negative effect on adDMN (*β* = −0.07, *P* = 0.002), pDMN (*β* = −0.27, *P* < 0.001) and vDMN (*β* = −0.11, *P* < 0.001) connectivity (Figs 2A and 3). The opposite effect was found for pDMN, vDMN and NFQ in Alzheimer’s disease, where the older atypical Alzheimer’s disease participants had higher pDMN (*β* = 0.18, *P* = 0.04) and vDMN (*β* = 0.20, *P* < 0.001) connectivity and lower NFQ (*β* = −0.36, *P* = 0.03) than the younger ones (Fig. 3). In amnestic Alzheimer’s disease, there was a significant positive effect of age only on vDMN connectivity (*β* = 0.14, *P* = 0.002) and a trend on pDMN connectivity (*β* = 0.14, *P* = 0.07; Fig. 3A and B). Among CU individuals, there was a significant positive interaction between age and time on pDMN (*β* = 0.10, *P* = 0.04) and vDMN (*β* = 0.06, *P* = 0.03) connectivity, with older individuals declining less over time than the younger ones (Fig. 3A and B). The opposite was found for vDMN in atypical (*β* = −0.14, *P* = 0.03) and amnestic (*β* = −0.20, *P* = 0.001) Alzheimer’s disease, with younger participants declining less in connectivity over time (Fig. 3B). Similarly, a trend was present in the interaction between age and time on the NFQ in atypical Alzheimer’s disease participants (*β* = 0.32, *P* = 0.09; Fig. 3C). At 65 years of age, there were trends for avDMN connectivity declining more (*β* = −0.11, *P* = 0.07) and for the NFQ increasing more (*β* = 0.21, *P* = 0.06) over 1 year in amnestic Alzheimer’s disease participants than in CU individuals (Figs 2B and 3C). Across all CU individuals and Alzheimer’s disease participants, male sex was associated with lower pDMN connectivity (*β* = −0.11, *P* = 0.01) and APOE ɛ;4 carriers had lower vDMN connectivity (*β* = −0.06, *P* = 0.05) and higher NFQ (*β* = 0.21, *P* = 0.03; Fig. 3). Covarying for fMRI maximum head motions and frame-wise displacement did not alter the statistical significance of the main findings of the mixed-effects models, except that the difference in NFQ between atypical Alzheimer’s disease patients and CU individuals was not significant anymore but only a trend.

#### PCA versus LPA

When the mixed-effects models were run on the atypical Alzheimer’s disease participants only, comparing PCA to LPA, at 65 years of age, LPA had higher avDMN connectivity compared to PCA (*β* = 0.17, *P* = 0.03), while PCA experienced a faster increase in avDMN connectivity over time (*β* = 0.15, *P* = 0.05). In LPA, pDMN connectivity declined more over time than in PCA (*β* = −0.31, *P* = 0.05). In PCA, there was a stronger positive effect of age on vDMN connectivity (*β* = 0.13, *P* = 0.04) and negative interaction of age and time on it (*β* = −0.13, *P* = 0.04).

#### Effect of tau in atypical Alzheimer’s disease

**Figure 4:**
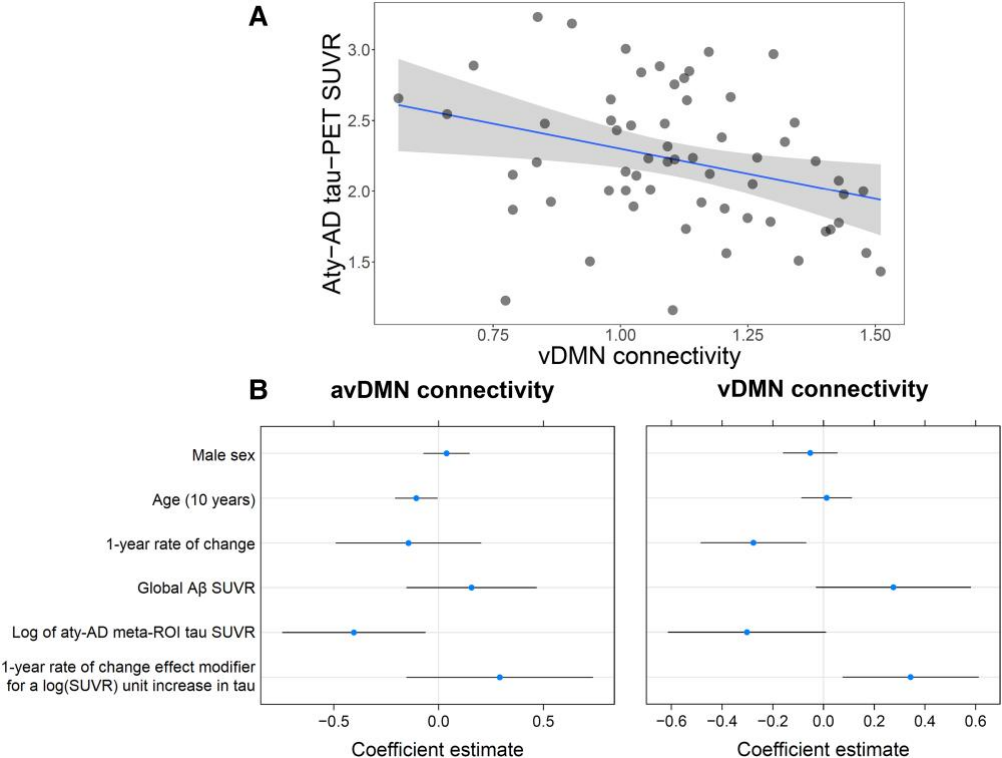
**Tau and functional connectivity in atypical Alzheimer’s disease.** Cross-sectional linear regression with 95% confidence intervals performed on atypical Alzheimer’s disease participants between vDMN connectivity *z*-scores and tau-PET SUVR calculated in the atypical Alzheimer’s disease meta-ROI (**A**). Longitudinal mixed-effects model estimates for avDMN and vDMN functional connectivity in atypical Alzheimer’s disease participants and tau-PET SUVR calculated in the atypical Alzheimer’s disease meta-ROI (**B**). Models are run including 61 atypical Alzheimer’s disease, 41 of which with follow-up data. Results are reported with age centred at 65 years. All fixed effects are reported with 95% CIs. Ninety-five per cent confidence intervals that do not cross the 0 line represent significant effects at the *P* < 0.05 level. Aty-AD, atypical Alzheimer’s disease; avDMN, anterior ventral default mode network; vDMN, ventral default mode network.

The only sub-network with a statistically significant cross-sectional association with tau was the vDMN, whose connectivity was negatively correlated with tau-PET SUVR calculated in the atypical Alzheimer’s disease meta-ROI (*β* = −0.71, adjusted *R*^2^ = 0.08, *P* = 0.01; Fig. 4A). In longitudinal mixed-effects models, higher baseline tau-PET SUVR was associated with lower avDMN connectivity (*β* = −0.40, *P* = 0.02) and there was a trend for a negative association between tau and vDMN connectivity (*β* = −0.30, *P* = 0.06; Fig. 4B). Time had a significant negative effect on vDMN connectivity (*β* = −0.27, *P* = 0.009), and there was a significant positive interaction between time and tau-PET SUVR (*β* = 0.34, *P* = 0.01), with vDMN connectivity declining more in participants with less tau, so possibly at a more preliminary disease stage (Fig. 4B). Baseline global Aβ did not have any significant effect on DMN sub-system connectivity or on NFQ. Including the atypical Alzheimer’s phenotype diagnosis as a fixed effect did not alter these results.

#### Clinical connectivity correlations in Alzheimer’s disease

When both amnestic and atypical Alzheimer’s disease participants were analysed together, vDMN connectivity was positively associated with MMSE scores (*β* = 5.0, adjusted *R*^2^ = 0.02, *P* = 0.05) and negatively associated with CDR-SB (*β* = −4.3, adjusted *R*^2^ = 0.07, *P* = 0.002); there was also a trend for the NFQ being associated with CDR-SB (*β* = 2.3, adjusted *R*^2^ = 0.02, *P* = 0.06) and for avDMN connectivity being associated with MMSE (*β* = 4.2, adjusted *R*^2^ = 0.02, *P* = 0.08). Atypical Alzheimer’s disease participants with worse simultanagnosia scores had lower avDMN (*β* = 7.9, adjusted *R*^2^ = 0.06, *P* = 0.04) and vDMN (*β* = 8.7, adjusted *R*^2^ = 0.06, *P* = 0.04) connectivity. Additionally, in atypical Alzheimer’s disease, there were trends for higher vDMN connectivity being associated with higher MoCA scores (*β* = 7.1, adjusted *R*^2^ = 0.04, *P* = 0.06), for higher adDMN connectivity being associated with higher VOSP letters scores (*β* = 7.1, adjusted *R*^2^ = 0.05, *P* = 0.06) and for higher avDMN (*β* = 4.1, adjusted *R*^2^ = 0.04, *P* = 0.07) and vDMN connectivity (*β* = 4.3, adjusted *R*^2^ = 0.03, *P* = 0.09) being associated with higher VOSP cube scores.

### Voxel-based analyses in atypical Alzheimer’s disease

#### Cross-sectional analyses

**Figure 5:**
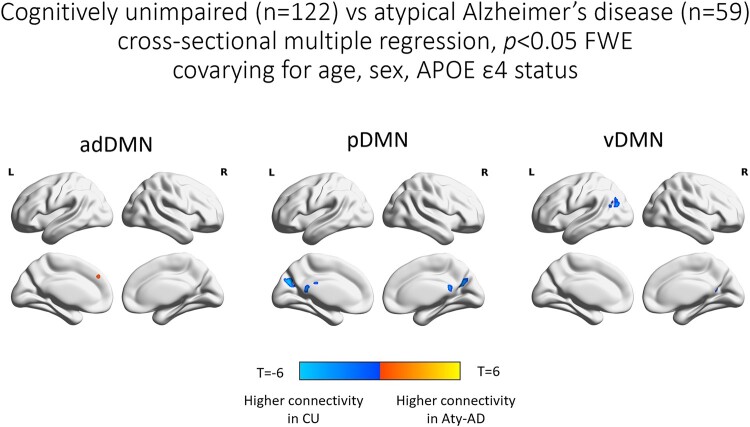
**Voxel-level maps in atypical Alzheimer’s disease.** SPM multiple regression results comparing adDMN, pDMN and vDMN connectivity between atypical Alzheimer’s disease participants (*n* = 59) and CU individuals (*n* = 122), covarying for age, sex and APOE ɛ;4 status. Results are reported at *P* < 0.05 with FWE correction for multiple comparisons and masked with the MCSA Functional Connectivity Atlas regions. Aty-AD, atypical Alzheimer’s disease; CU, cognitively unimpaired; adDMN, anterior dorsal default mode network; pDMN, posterior default mode network; vDMN, ventral default mode network.

Connectivity maps of each DMN sub-network from one-sample *t*-tests within atypical Alzheimer’s disease participants and CU individuals are reported as supplemental (Supplementary Fig. 1). Voxel-based analyses confirmed the findings from mixed-effects models on regional connectivity values, with some differences due to modelling discrepancies (Fig. 5; Supplementary Figs 2 and 3). Covarying for age, sex and APOE ɛ;4 status, atypical Alzheimer’s disease participants had decreased pDMN and vDMN connectivity and increased adDMN connectivity relative to CU individuals, at *P* < 0.05 with family-wise error (FWE) correction for multiple comparisons (Fig. 5). At *P* < 0.001 without correction for multiple comparisons, there was evidence of decreased connectivity within the avDMN regions in atypical Alzheimer’s disease relative to CU individuals (Supplementary Fig. 2A). When PCA and LPA participants were analysed separately against age- and sex-matched CU individuals, covarying for age, sex and APOE ɛ;4 status, PCA participants showed increased adDMN connectivity and decreased avDMN, pDMN and vDMN connectivity relative to CU individuals, at *P* < 0.001 without correction for multiple comparisons (Supplementary Fig. 2B). The lower vDMN connectivity in PCA participants relative to CU individuals survived FWE correction for multiple comparisons at *P* < 0.05. LPA participants showed lower avDMN and pDMN connectivity and higher adDMN and vDMN connectivity relative to age- and sex-matched CU individuals, with lower pDMN and higher adDMN surviving FWE correction for multiple comparisons at *P* < 0.05 (Supplementary Fig. 2C).

#### Longitudinal analyses

At *P* < 0.001 without correction for multiple comparison, there was no evidence of connectivity change over time on paired *t*-tests between baseline and follow-up images in CU individuals (Supplementary Fig. 3A), but there was evidence of vDMN connectivity decline in atypical Alzheimer’s disease participants, which did not survive FWE correction for multiple comparisons at *P* < 0.05 (Supplementary Fig. 3B).

## Discussion

We observed that cross-sectional and longitudinal patterns of DMN sub-system connectivity differ between the language (i.e. LPA) and visual (i.e. PCA) atypical variants of Alzheimer’s disease and age-matched CU individuals. We also found differences in DMN connectivity between the two atypical Alzheimer’s phenotypes. Not all DMN sub-systems exhibited the same trends: while the posterior regions had lower connectivity in atypical Alzheimer’s disease, the opposite was found in some of the anterior regions of the network. Age-matched amnestic Alzheimer’s disease participants exhibited analogous patterns. Age had a negative effect on DMN functional connectivity in CU individuals (i.e. older CU individuals had lower connectivity than younger ones) and a positive effect in Alzheimer’s disease participants (i.e. younger Alzheimer’s disease participants had more disrupted connectivity than older ones relative to CU individuals). The vDMN was the only sub-system to show connectivity decline over 1 year in atypical Alzheimer’s disease, particularly in older participants. In atypical Alzheimer’s disease participants, tau had a detrimental effect on functional connectivity within the avDMN and vDMN. Connectivity within the avDMN and vDMN sub-systems was also associated with visuospatial cognition, namely with the simultanagnosia score, in atypical Alzheimer’s disease.

There was a striking effect of age on default mode sub-network connectivity, which was more abnormal in younger atypical Alzheimer’s disease participants relative to CU individuals, similarly to other Alzheimer’s disease biomarkers, particularly tau-PET uptake.^25,27,49,50^ Specifically, in mixed-effects models, pDMN and vDMN were significantly lower and NFQ was significantly higher in atypical Alzheimer’s disease participants than in CU at ages 55 and 65, but not at age 75. The amnestic variant showed an analogous, although less striking, positive effect of age on DMN, which was significant only for vDMN connectivity. The current results for the NFQ in the visual and language variants of Alzheimer’s disease mirror analogous findings in the executive variant of Alzheimer’s disease, confirming its ability to discriminate between Alzheimer’s disease variants and CU individuals.^6,12^

Connectivity within the pDMN was significantly reduced in the visual and language variants of Alzheimer’s disease, both on regional mixed-effects models and voxel-level analyses, unlike in a previous report on these two syndromes.^13^ This discrepancy could depend on differences in age and disease stage or on differences in pre-processing and analyses of the functional images. We defined networks from independent component analysis on a large population of CU individuals,^9^ while the previous report used a seed-based approach.^13^ Although pDMN connectivity was lower cross-sectionally in atypical Alzheimer’s disease participants relative to CU individuals, the models did capture a decline over time. However, when PCA and LPA were compared directly to each other, LPA showed a faster decline in pDMN connectivity over time than PCA. This difference between the two variants could be a consequence of the less advanced disease stage in LPA participants than in PCA. These observations fit with the cascading network failure model, which identifies the decline in pDMN connectivity as an early feature of Alzheimer’s disease.^10^ There were no differences in pDMN connectivity between PCA and LPA participants: both phenotypes showed lower connectivity relative to age-matched CU individuals, mirroring the age-matched amnestic participants. This finding supports the central hypothesis of the cascading network failure model, which is that pDMN failure is common across Alzheimer’s disease phenotypes, and it interacts with pre-existing phenotype-specific network vulnerabilities (i.e. medial temporal lobe in amnestic Alzheimer’s disease, left temporal language network in LPA and occipital visual network in PCA).^11^

Like pDMN, vDMN connectivity was lower in both amnestic and atypical Alzheimer’s disease participants than in CU individuals, both on regional mixed-effects models and voxel-level analyses. However, in general, the ability of voxel-based analyses to capture connectivity differences was more limited, with only small clusters of voxels surviving correction for multiple comparisons. Within the atypical participants, the finding of disrupted vDMN connectivity was driven by the PCA participants. Unlike pDMN connectivity, vDMN connectivity differed between PCA and LPA: while the regional mixed-effects model did not find any statistically significant differences in the direct comparison of vDMN connectivity between PCA and LPA, when the two phenotypes were compared separately to age-matched CU individuals with voxel-based analyses, only PCA exhibited lower vDMN connectivity. Instead, vDMN connectivity was slightly increased in LPA participants alone relative to CU individuals, but this finding did not survive correction for multiple comparison, consistent with what our group reported in a previous study.^14^ The vDMN overlaps with the dorsal attention network, particularly relevant in the visual variant of Alzheimer’s disease,^51,52^ providing a potential explanation for this sub-network being disrupted only in PCA. However, it is hard to say to what extent the difference in vDMN connectivity between PCA and LPA was phenotype driven as opposed to a consequence of disease stage (shorter in LPA) and age (higher in LPA) differences. Given the positive effect of age on vDMN connectivity in atypical Alzheimer’s disease, it is reasonable to assume that the more intact vDMN in the LPA participants could be in part explained by their older age relative to PCA.

Time did not have a strong effect on DMN sub-system connectivity. This could be a consequence of the short follow-up time and the advanced disease stage of the patient cohorts, as DMN changes are tightly related to amyloidosis,^11,22^ which does not significantly increase over time at this stage. Also, the inherent instability of fMRI measures poses challenges in detecting within-person changes over 1 year. Nevertheless, vDMN connectivity exhibited a decline over 1 year in atypical Alzheimer’s disease participants that was not detected in age-matched CU individuals. This decline was faster in older Alzheimer’s participants, and the same negative interaction between time and age on vDMN connectivity was present in amnestic participants. The fact that longitudinal decline in vDMN connectivity was slower in younger Alzheimer’s participants contrasts with observations on rates of tau accumulation and rates of grey matter atrophy, which are faster in younger atypical Alzheimer’s disease participants,^25^ putting DMN connectivity on a different longitudinal course than these two other biomarkers. Age and time interacted on vDMN connectivity in opposite ways among CU individuals and both amnestic and atypical Alzheimer’s disease participants. At a younger age, CU individuals declined faster over time than atypical Alzheimer’s disease participants, while at an older age, it was atypical Alzheimer’s disease participants who declined faster over time than CU individuals. This contrasts with a previous study that reported an acceleration of DMN connectivity decline after age 74, preceded by an increase in DMN connectivity between ages 50 and 66.^2^ Our cohort likely did not include enough individuals older than 75 years of age to capture this effect. Additionally, the previous study investigated only healthy controls, without Alzheimer’s pathology.^2^

Anterior DMN connectivity is typically increased in Alzheimer’s disease compared to healthy controls,^5,10,53^ as connectivity shifts from the posterior regions. We found that connectivity within the adDMN was increased in both atypical and amnestic Alzheimer’s disease relative to the CU individuals, when age was centred at 65 or 75 years, and not at 55, hinting that the increase in anterior dorsal connectivity may reflect a compensatory phenomenon after the decrease in posterior connectivity. In a previous study, typical amnestic Alzheimer’s participants showed higher avDMN connectivity and not higher adDMN connectivity.^10^ A reason for this discrepancy could be that the amnestic cases included in the current study were age matched with the atypical cases and therefore most would fall into the ‘early-onset’ category. Connectivity in the avDMN sub-system differed between PCA and LPA and, as vDMN, was more disrupted in PCA. Unlike for vDMN, age did not have a significant positive effect on avDMN connectivity in atypical Alzheimer’s disease relative to CU individuals. Therefore, it is likely that the longer disease duration in PCA than in LPA, more than the younger age, could in part explain the less disrupted avDMN connectivity in LPA. Assuming that tau spreads trans-synaptically and impairs functional connectivity,^19,51^ the current finding of a less disrupted avDMN and vDMN connectivity in LPA fits with our recent report that, in a partially overlapping cohort, LPA participants exhibited significantly faster rates of tau-PET uptake accumulation across the cortex than PCA.^54^ In addition, avDMN and vDMN were the two sub-systems whose connectivity was negatively associated with tau burden in atypical Alzheimer’s disease participants. The avDMN does not overlap with the atypical Alzheimer’s disease meta-ROI used for calculating tau-SUVR, but it covers frontal regions that undergo tau accumulation over time in PCA and LPA, with faster rates in case of higher baseline parietal tau burden.^25^ Tau burden also played a role on vDMN connectivity change over time, with higher tau SUVR in the atypical Alzheimer’s disease meta-ROI being associated not only with lower connectivity but also with more moderate connectivity decline over 1 year. Regions of the vDMN, including the precuneus, posterior cingulate cortex and angular gyrus, have substantial tau pathology in PCA and LPA,^25^ and tau impairs functional connectivity, hence the decline in vDMN connectivity over time. On the other hand, within the atypical Alzheimer’s disease participants, global amyloid-β SUVR was not associated with DMN sub-system connectivity. This was not surprising since amyloid-β levels do not usually relate to other disease biomarkers at advanced disease stage, as in the case of the participants of this study. In the Alzheimer’s disease spectrum, amyloid-β did not correlate with lower pDMN connectivity but with higher pDMN to vDMN connectivity^10^ and disrupted DMN–salience connectivity over time.^22^

Among cognitively normal individuals, APOE ɛ;4 carriers have been reported to have increased DMN connectivity at young age^55^ and decreased pDMN connectivity at older age,^10,56^ without alteration in vDMN connectivity relative to non-carriers.^10^ Within both atypical and amnestic Alzheimer’s disease participants and CU individuals, we found lower vDMN connectivity and higher NFQ in APOE ɛ;4carriers.

Worse general cognitive function and worse clinical severity were related to lower vDMN connectivity within amnestic and atypical Alzheimer’s disease participants analysed together and, less strongly, within the atypical phenotypes alone.^10,19^ Instead, no associations were found between pDMN connectivity and cognitive performances. There was some evidence of DMN connectivity being associated to visuospatial symptoms in atypical Alzheimer’s disease, particularly simultanagnosia, in agreement with a recent study from our group.^15^ The vDMN sub-system overlaps with the dorsal attention network in the parietal cortex, whose connectivity has been related to visuospatial symptoms in PCA.^51^ In our cohort of both PCA and LPA, visuospatial symptoms were also related to the avDMN. If for the correlation between vDMN connectivity and visuospatial symptoms we can hypothesize a causal relationship, the correlation with avDMN may be driven by the concurrent lower avDMN connectivity and worse visuospatial performances in PCA participants. The somewhat limited associations between DMN connectivity and atypical Alzheimer’s disease symptoms are not surprising since PCA and LPA symptoms are more specific to brain regions in other functional networks, like the visual, dorsal attention or language networks. Again, this finding fits with the cascading network failure model that postulates that failure in posterior DMN regions is common across Alzheimer’s disease phenotypes, and it interacts with pre-existing phenotype-specific network vulnerabilities.^10,11^

Functional connectivity was analysed without covarying for grey matter volume. Although it is plausible that loss of grey matter influences functional connectivity, we made the choice not to regress it out because grey matter atrophy is a measure of the disease effect, which was the very thing we aimed to investigate in the context of DMN functional connectivity. Furthermore, it was previously reported that DMN connectivity changes in Alzheimer’s disease survive correction for grey matter atrophy.^5,21^ Additionally, the short time distance between baseline and follow-up scans helps mitigate the effect of atrophy on changes in functional connectivity.^21^

The main strengths of this study are the analysis of longitudinal resting-state fMRI for two relatively rare atypical Alzheimer’s disease variants in comparison to the amnestic variant and the use of networks derived from independent component analysis performed on a large population of healthy CU individuals,^9^ which may be more stable than seed-based connectivity and therefore more suited for intra-individual longitudinal analyses.^57^ The main limitation is the availability of only two timepoints, which prevented us from modelling the non-linear effect of age on DMN functional connectivity.^2^ A secondary limitation is the age and disease duration difference between PCA and LPA participants, which might have influenced the difference in connectivity between them.

In summary, spatiotemporal trajectories of abnormal DMN connectivity were observed in the visual and language variants of Alzheimer’s disease, with alterations relative to CU individuals being more pronounced in younger participants and mirroring the patterns observed in the amnestic variant. These findings highlight the key role that the DMN plays in Alzheimer’s disease pathophysiology across clinical phenotypes, making it a diagnostic biomarker and a potential target for intervention, with distinction between anterior and posterior sub-networks.

## Supplementary Material

fcae005_Supplementary_Data

## Data Availability

Data that support the findings of this study are available from the corresponding author upon reasonable request.
