# Supplementary material for: Longitudinal default mode sub-networks in the language and visual variants of Alzheimer’s disease
Source: Brain Commun. 2024 Jan 8;6(2):fcae005. doi: 10.1093/braincomms/fcae005 (PMC10914456; doi:10.1093/braincomms/fcae005)

**Supplemental Figure 1**. DMN sub-networks connectivity maps from one sample *t*-tests on atypical Alzheimer’s disease participants (aty-AD) (**A**) and cognitively unimpaired individuals (CU) (**B**). Results are reported at *p*<0.05 with FWE correction for multiple comparisons. CU = cognitively unimpaired. aty-AD = atypical Alzheimer’s disease. adDMN = anterior dorsal default mode network. avDMN = anterior ventral default mode network. pDMN = posterior default mode network. vDMN = ventral default mode network.


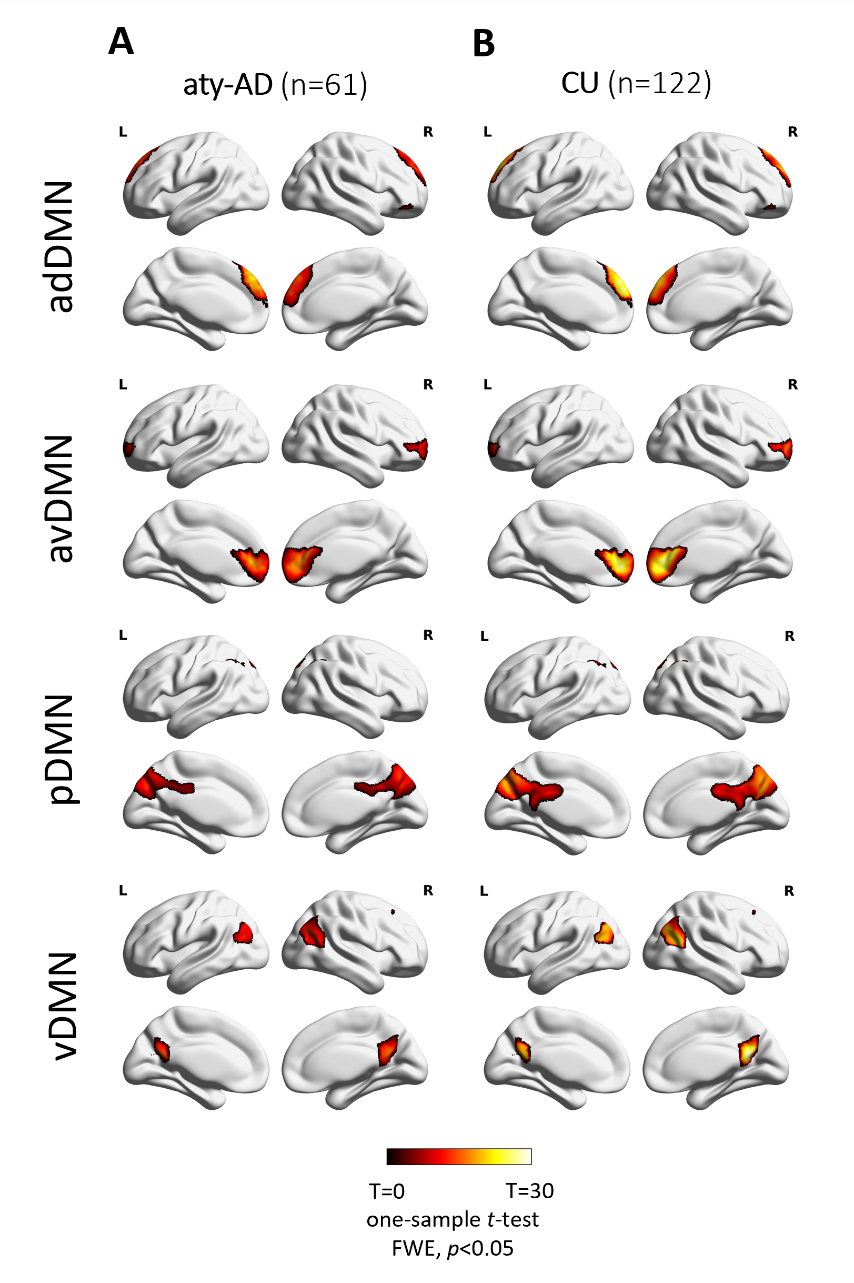


**Supplemental Figure 2**. SPM multiple regression results for the comparison of adDMN, avDMN, pDMN, vDMN connectivity between all atypical Alzheimer’s participants (n=59) (**A**), participants with the visual variant (PCA, n=32) (**B**) and participants with the language variant (LPA, n=27) (**C**) and age- and sex-matched cognitively unimpaired individuals, covarying for age, sex, and APOE ε4 status. Results are reported at *p*<0.001 uncorrected for multiple comparison and masked with the MCSA Functional Connectivity Atlas regions. The color bar represents the T-statistic, and the vertical black bar indicates the T-value cut-off for FWE correction for multiple comparisons at *p*<0.05 in each cohort. CU = cognitively unimpaired. aty-AD = atypical Alzheimer’s disease. PCA = posterior cortical atrophy. LPA = logopenic progressive aphasia. adDMN = anterior dorsal default mode network. avDMN = anterior ventral default mode network. pDMN = posterior default mode network. vDMN = ventral default mode network.

**
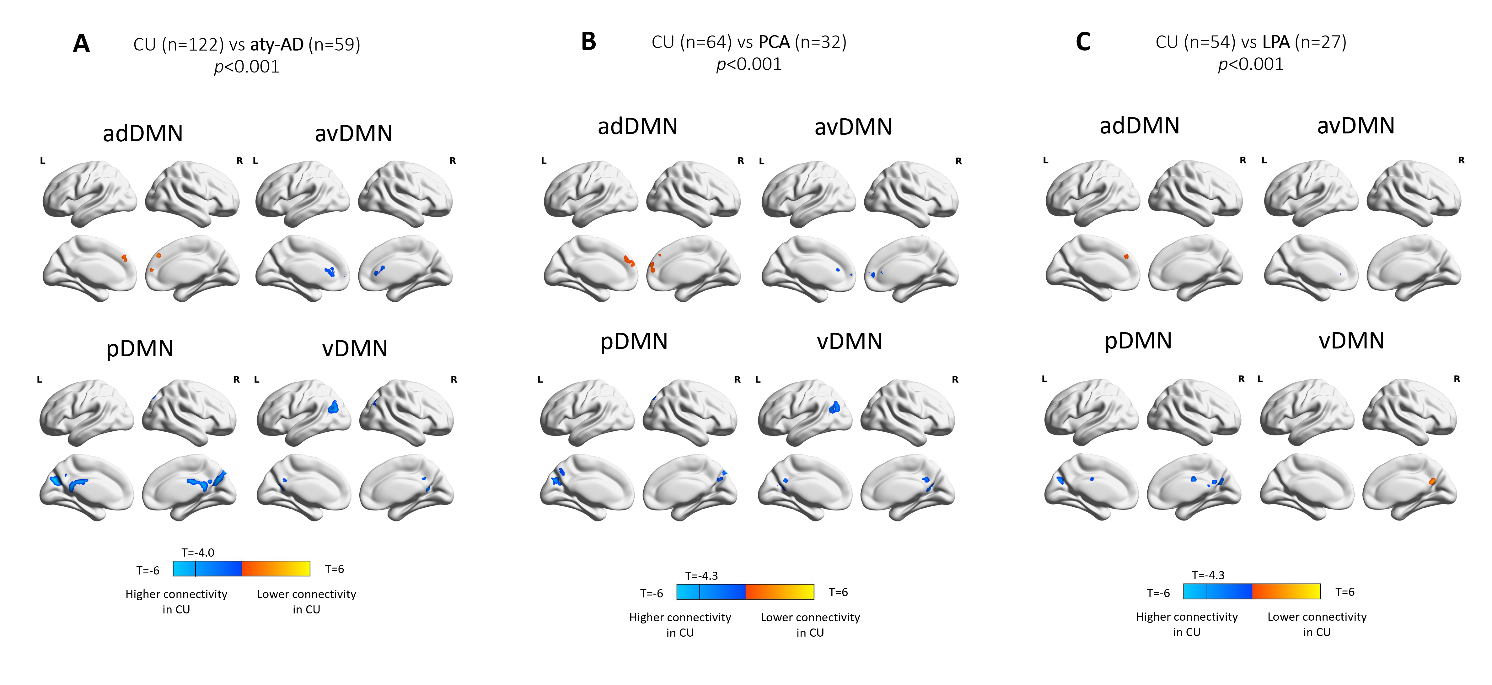
**

**Supplemental Figure 3**. SPM paired *t*-tests results of the comparison between baseline and follow-up scans of adDMN, avDMN, pDMN, and vDMN connectivity in cognitively unimpaired individuals (n=40) (**A**) and atypical Alzheimer’s disease participants (n=41) (**B**). Results are reported at *p*<0.001 uncorrected for multiple comparison and masked with the MCSA Functional Connectivity Atlas regions. The color bar represents the T-statistic. CU = cognitively unimpaired. Aty-AD = atypical Alzheimer’s disease. adDMN = anterior dorsal default mode network. avDMN = anterior ventral default mode network. pDMN = posterior default mode network. vDMN = ventral default mode network.


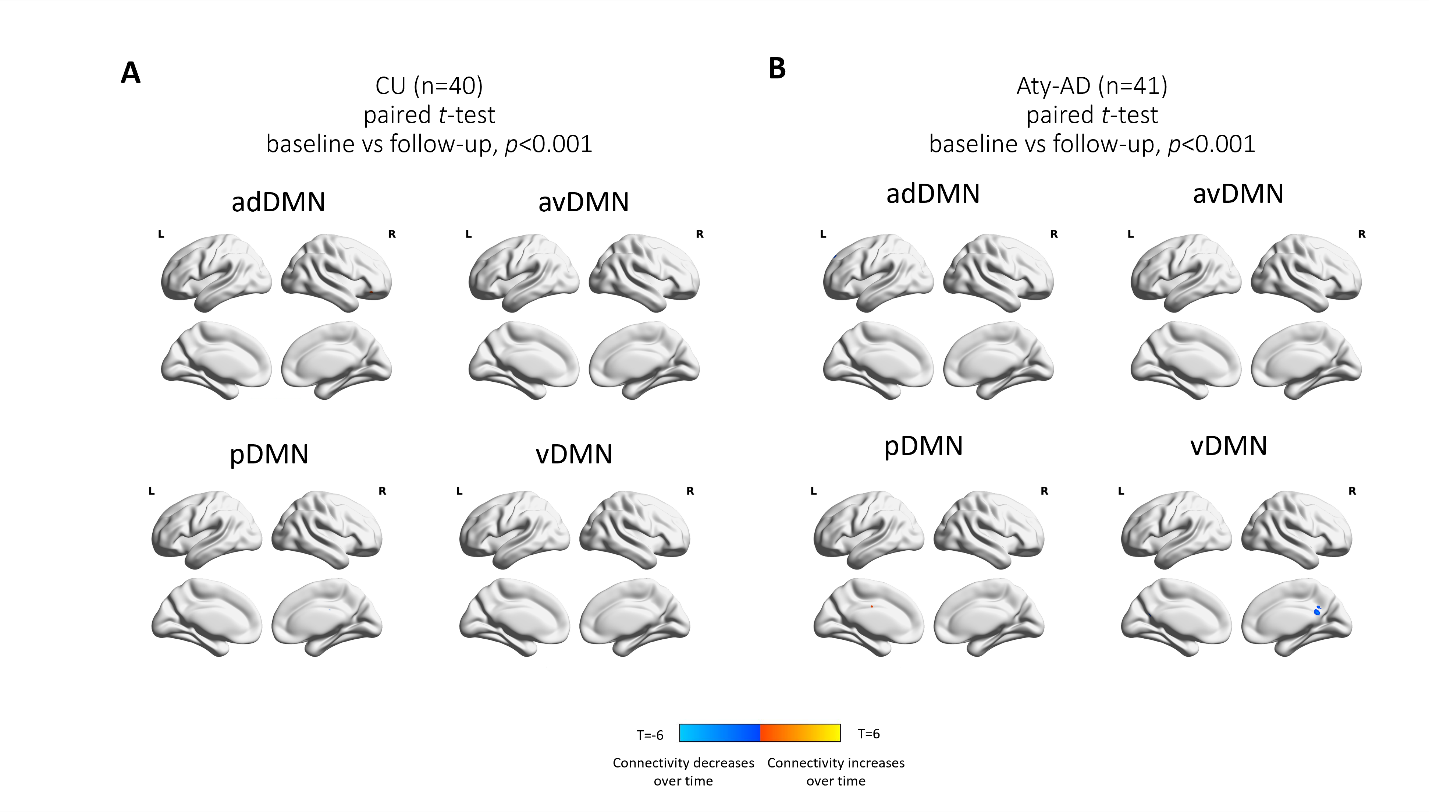

Supplement: fcae005_Supplementary_Data [file fcae005_supplementary_data.docx]
